# Interindividual neural differences in moral decision-making are mediated by alpha power and delta/theta phase coherence

**DOI:** 10.1038/s41598-019-40743-y

**Published:** 2019-03-14

**Authors:** Annemarie Wolff, Javier Gomez-Pilar, Takashi Nakao, Georg Northoff

**Affiliations:** 1 Institute of Mental Health Research, University of Ottawa, Ottawa, Canada; 2 Biomedical Engineering Group, Higher Technical School of Telecommunications Engineering, University of Valladolid, Valladolid, Spain; 3 Department of Psychology, Graduate School of Education, Hiroshima University, Hiroshima, Japan

## Abstract

As technology in Artificial Intelligence has developed, the question of how to program driverless cars to respond to an emergency has arisen. It was recently shown that approval of the consequential behavior of driverless cars varied with the number of lives saved and showed interindividual differences, with approval increasing alongside the number of lives saved. In the present study, interindividual differences in individualized moral decision-making at both the behavioral and neural level were investigated using EEG. It was found that alpha event-related spectral perturbation (ERSP) and delta/theta phase-locking – intertrial coherence (ITC) and phase-locking value (PLV) – play a central role in mediating interindividual differences in Moral decision-making. In addition, very late alpha activity differences between individualized and shared stimuli, and delta/theta ITC, where shown to be closely related to reaction time and subjectively perceived emotional distress. This demonstrates that interindividual differences in Moral decision-making are mediated neuronally by various markers – late alpha ERSP, and delta/theta ITC - as well as psychologically by reaction time and perceived emotional distress. Our data show, for the first time, how and according to which neuronal and behavioral measures interindividual differences in Moral dilemmas can be measured.

## Introduction

### Interindividual differences in Moral preference – driverless cars illustrate its relevance

Imagine you are riding in a driverless car. The car encounters a crossing, but for whatever reason cannot stop. Should the car sacrifice you, the passenger? Or should it sacrifice the ten pedestrians on the sidewalk? What would you as the owner of the car prefer? This Moral dilemma, previously a philosophical ‘thought experiment’^1^, has become an urgent public policy concern.

As of April 2, 2018, the state of California has allowed driverless cars on its roads; prior to that date, an approved person was required to sit in the driver seat to take control in case of emergency^2^. With their arrival in our daily routines, the practicalities of driverless cars will add a layer of complexity to previously studied Moral dilemmas, specifically the Trolley and Footbridge problems^1^.

Imagine you then pose the same questions to your friends and colleagues. They provide you with a variety of responses. The point on which there is the most divergence is the number of pedestrians for which they would be willing to sacrifice themselves as passengers. Two recent investigations showed that most participants prefer these consequentialist decisions – minimizing the harm by maximizing the number of people saved - in what the authors describe as an ‘emotionally salient’ situation^3,4^. In addition, approval of the consequential behavior of the driverless car varied with the number of lives saved. Approval increased with the number of lives saved doing likewise, but reached a maximum approval of 76% of participants. This, and other studies^5–7^, demonstrate the central relevance of interindividual differences in Moral decision-making.

These interindividual differences revealed that participants are more consequential; they approve of actions in which the difference in numbers of saved to killed is small. Some, on the other hand, responded in a less consequential manner in that the ratio was larger. As other studies on Moral dilemmas show, the subjectively perceived emotional distress of the participant has a significant effect on their response^8,9^ and reaction time^9^.

What, though, are the behavioral and neural bases for this interindividual variability in Moral preference? Many recent neural and psychological studies have examined ethical dilemmas^10–15^. None, however, have examined and tested explicitly for interindividual differences, at the behavioral and neural levels, within consequential scenarios in which the number of killed to saved varied. The closest study would be Chen^16^ which varied the relationships of participants in the scenarios within the relevant to participants’ context of an earthquake. They found a higher amplitude of the P300 when the stress was increased when relatives were involved in the dilemma than when the other individuals were strangers. Interindividual variation of the Moral paradigms and their neural and behavioral effects remain unclear in current neuroscience. The aim of the current study, therefore, was to explicitly investigate the neural and behavioral correlates of interindividual differences in Moral preference.

### Moral decision-making – Neural correlates of interindividual differences

Much research has been done on the behavioral, psychological and neurophysiological responses to the iconic ‘Footbridge’ and ‘Trolley’ Moral dilemmas of Phillipa Foot^1,7,8,10,11,15,17–22^, though most of this is comprised of fMRI imaging. These studies have found that areas associated with emotional processing such as the medial frontal gyrus, the posterior cingulate gyrus and the bilateral angular gyri are more active during Footbridge-like moral dilemmas than during Trolley-like dilemmas and non-moral dilemmas, while areas associated with working memory, such as the right middle frontal gyrus and the bilateral parietal lobes, are less active^17,18^.

In recent years, some researchers have investigated the temporal dynamics of Moral reasoning using electroencephalography (EEG) and event-related potentials (ERPs)^12–15,23,24^.

**Figure 1:**
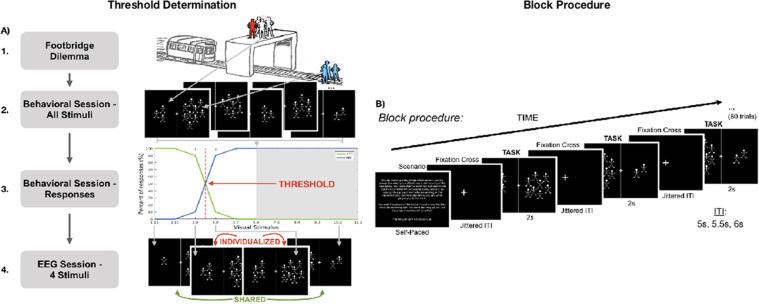
Study threshold determination and block procedure. (**A**) Determination of threshold in behavioral session. 1 Participants read a Footbridge-type dilemma in the behavioral session. *Red*: The participant in the scenario; *White*: the people that they would kill in the scenario; *Blue*: the people that would be saved in the scenario because they killed the others. 2 Participants were presented with 10 repetitions of each stimulus in a randomized order. 3 Based on the percentage of YES and NO responses for each stimulus in the preceding session, the threshold (dashed red vertical line) was calculated. 4 Finally, the stimuli immediately below and above the threshold - the Individualized stimuli - as well as two other stimuli Shared by all participants - 1:11 and 10:2 - were the only stimuli presented to participants in the subsequent EEG session. (**B**) Block procedure for the behavioral and EEG session. The participant was presented one of three Footbridge-type dilemmas, which they read at their own pace. The trials were preceded by a fixation cross for a jittered duration of either 5, 5.5 or 6 seconds (randomized). The stimuli were a black screen with white two-dimensional stick people on either side of the screen, with a white line and fixation cross down the middle. The instructions given were that the people on the left side were those that would be killed in the previously read scenario, while the people on the right were those that were saved because of the others dying. Participants were instructed to respond either YES or NO if the numbers of killed to saved presented in the stimulus were acceptable to them. The maximum duration of the stimulus was 2 seconds. In the EEG session, each block was comprised of 60 trials, with each stimulus being presented 15 times. ITI = intertrial interval.

A high temporal resolution may be central for detecting interindividual differences in Moral preference which manifest in the time course of neural activity. For this reason we chose EEG to investigate Moral preference^7,12–15^. Most importantly, using EEG we examined interindividual differences through the individualizing of stimuli as well as through variation of the degree of consequentialism. The degree of consequentialism was varied by presenting participants with different ratios of killed to saved (one killed to save many, several killed to save many, number of people saved is only one more than killed, etc (Fig. 1A-1).

### Aims and hypotheses

The main and overarching aim of our study was to investigate neural and behavioral correlates of interindividual differences in Moral preference. For that purpose, we designed a specific paradigm (Fig. 1A) that, as based on the well-known Footbridge dilemma^1,17–20,25^, allowed us to test interindividual differences in response to individually-tailored degrees of consequentialist decision-making.

Our specific aims were threefold. First, we examined the difference between the Moral and Control conditions, as well as between stimuli with varied ratios, through behavioral and ERP data. We hypothesized that late components^7,13,16,26^, specifically the Late Positive Potential (LPP), are related to interindividual differences in Moral preference as they are closely related to the processing of emotions^27–32^ which are core features of Moral preference^12,33–35^.

Secondly, in later ERP time intervals found to be have significant differences, differences in frequency band power were investigated, while phase coherence related to the stimuli was investigated in the full epoch. While no studies have investigated these neuronal measures in moral paradigms, several studies have demonstrated the central role of alpha ERSP and delta/theta ITC in mediating interindividual differences^36–39^. We therefore hypothesized that alpha event-related spectral perturbation (ERSP) and intertrial coherence (ITC) in the delta/theta range would be central in mediating interindividual differences in Moral preference.

Finally, the relationship between the behavioral and the neural activity related to individualized and shared stimuli, frequency band power, and phase coherence will be determined. We hypothesized that interindividual differences in neuronal measures – LPP in individualized stimuli, alpha ERSP, and delta/theta ITC - during Moral decision-making are related to interindividual differences in both reaction time and perceived emotion. From this, we reasoned that interindividual differences in Moral preference are closely related to emotional arousal/perception as well as in their reaction time.

## Results

### Reaction times were slower in the Moral condition and Individualized stimuli

**Figure 2:**
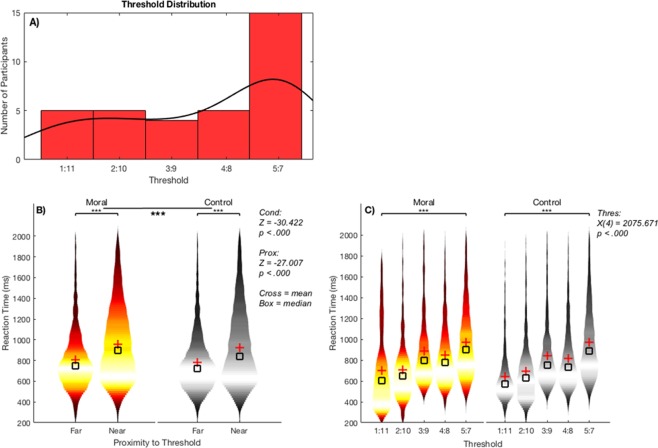
Threshold distribution and reaction time comparison between Moral and Control blocks in all trials. (**A**) Distribution of thresholds of all participants. There was a roughly even distribution of participants in the lowest four thresholds, while the highest threshold, 5:7, contained the largest number. (**B**) All trials in two Wilcoxon signed-rank tests measuring the effect of condition (Moral, Control) and proximity to threshold (Near, Far) on reaction time. There was a significant effect of both condition and proximity (*Z*- and *p*-values stated). (**C**) All trials in a Kruskal-Wallis H test (5 levels) measuring the effect of threshold (1:11, 2:10, 3:9, 4:8, 5:7) on reaction time. *Box* = median, *Cross* = mean.

The behavioral data showed significant differences in reaction time in the Moral condition and in the Individualized stimuli near the threshold in all trials. A Wilcoxon signed-rank test showed that condition (Moral, Control) had a statistically significant effect on the reaction times in all trials (*Z* = −30.422, *p* < 0.000) (Fig. 2B). Next, a Wilcoxon signed-rank test showed that proximity to threshold (Near Threshold, Far from Threshold) had a statistically significant effect on the reaction times in all trials (*Z* = −27.007, *p* < 0.000) (Fig. 2B). Indeed, mean Near Threshold reaction time was 902 ms while the mean for Far from Threshold stimuli was 763 ms.

Finally, a Kruskal-Wallis H test showed that there was a statistically significant effect of threshold on reaction time in all trials, χ^2^(4) = 2075.671, *p* < 0.000, with mean rank scores of the following: 1:11 = 3125 ms; 2:10 = 3909 ms; 3:9 = 5610 ms; 4:8 = 5359 ms; 5:7 = 6806 ms (Fig. 2C).

Therefore, reaction times were slower for the Moral condition, for Individualized stimuli, and in the more consequential thresholds.

### Event-related potentials (ERP’s) showed higher late activity in Individualized stimuli

The event-related potential (ERP) analysis found a significant effect of proximity to threshold in the N100, P300 and the LPP late time interval. For each time interval, a 2 (Moral, Control) x 2 (Near, Far) repeated measures ANOVA was performed on the maximum amplitude in the N100, N200 and P300 and the mean amplitude in the LPP.

**Figure 3:**
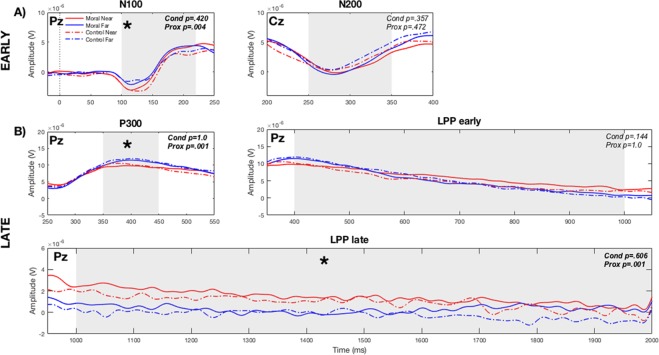
Event-related potentials (ERPs) of both early and late components. (**A**) Early components. In the individual plots the data from Pz for N100 and Cz for N200 (see Methods) for each peak is illustrated, with *p*-values for condition and proximity to threshold stated. N100 had a significant effect of proximity, while neither condition nor proximity were significant for N200. (**B**) Late components (at Pz). The P300 and the two time intervals for the LPP, with *p*-values for condition and proximity to threshold, are stated. In the P300, there was a significant effect of proximity to threshold, which was also true in the LPP late time interval. *P*-values stated.

In the early components, there was a significant main effect of proximity to threshold (Near, Far) in the N100 only (Wilks’ Lambda = 0.747, *F*(1,30) = 9.816, *p* = 0.004), with the Near Threshold stimuli having higher peaks than stimuli Far from the Threshold (Fig. 3A). There was no significant effect of condition (Moral, Control) in either the N100 or N200 (see Sup Mats).

For the late components, there were significant effects of proximity to threshold (Near, Far) in the P300 (Wilks’ Lambda = 0.675, *F*(1,30) = 13.964, *p* = 0.001) – higher peak amplitude in stimuli Far from the Threshold - and LPP late (Wilks’ Lambda = 0.701, *F*(1,30) = 12.395, *p* = 0.001) time intervals (Fig. 3B) which showed higher mean activity in stimuli Near the Threshold. In the LPP early time interval, there was a significant interaction between condition (Moral, Control) and proximity to threshold (Near, Far), (Wilks’ Lambda = 0.858, *F*(1,30) = 4.795, *p* = 0.037).

In sum, activity from Individualized stimuli was higher than Shared in both early (N100) and late (LPP late) ERP components but lower than shared in the P300.

### Moral alpha differed in event-related spectral perturbation (ERSP)

A significant difference in alpha (7–13 Hz) band power was found in the LPP time interval between Near Threshold and Far from Threshold in the Moral condition only.

**Figure 4:**
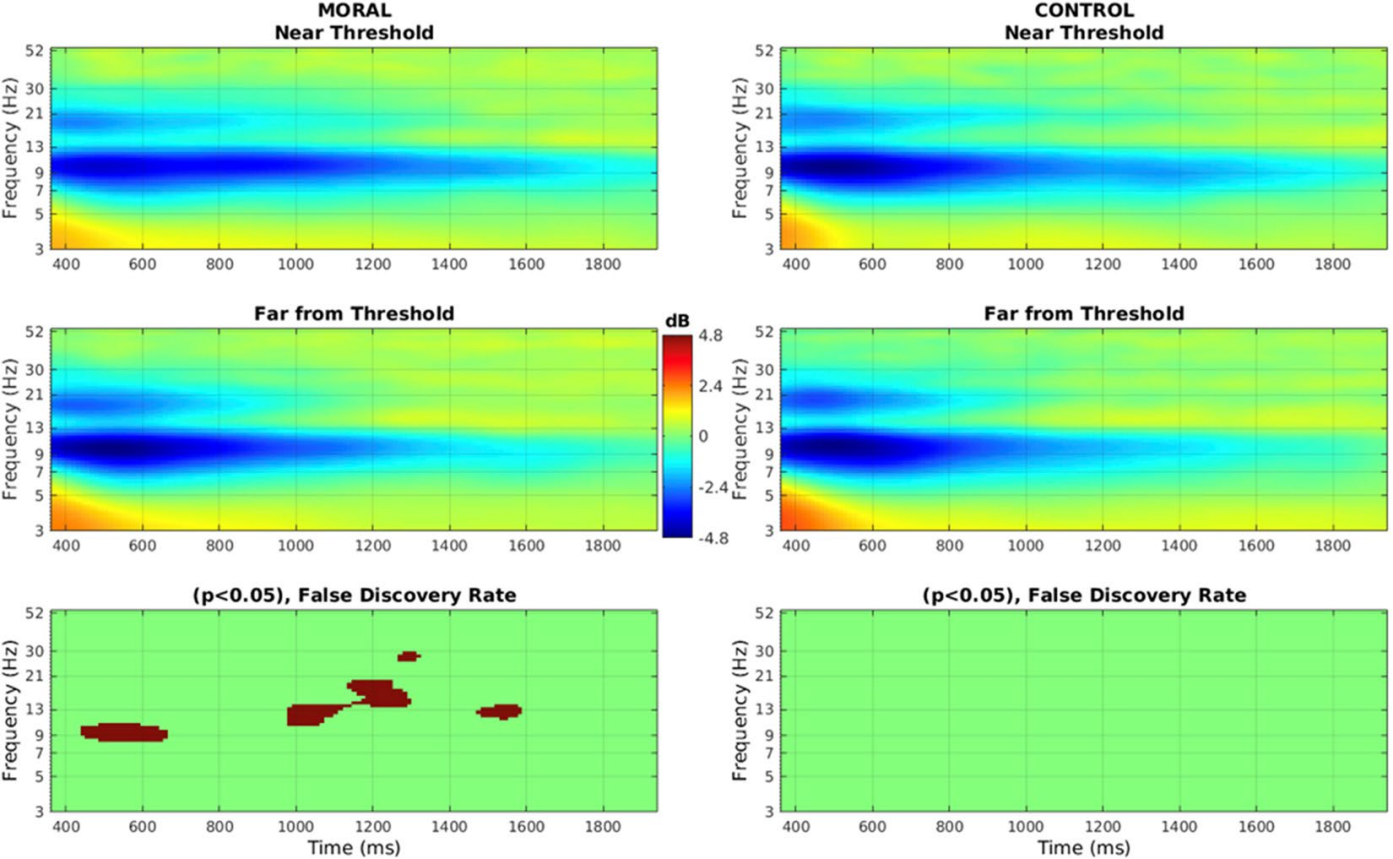
Event-related spectral perturbation (ERSP) at Pz of late component in both conditions and for both proximities to threshold. Compared to baseline, there was a significant difference in Alpha (7–13 Hz) and Low Beta (13–20 Hz) in the Moral condition (left side) between the stimuli Near Threshold and Far from Threshold. In the same stimuli, there was no significant difference in the Control condition (right side). The significance level is 0.05, and *t*-tests are corrected for multiple comparisons using the False Discovery Rate.

A paired-samples *t*-test, with the False Discovery Rate correction and a significance level of 0.05, showed a significant difference for the following times: between 8.2–11 Hz from 430 to 680 ms, 10.7–13.6 Hz from 970 to 1000 ms, 9.2–13.3 Hz from 1000 to 1136 ms, and 10.8–13.3 Hz from 1471 to 1600 ms (Fig. 4). To ensure that the response activity had no effect on these findings, an ERSP time-locked to the response in each trial was done (Sup Fig. 4). There was no significant difference between conditions related to response.

In sum, in the Moral condition there was a significant decrease in alpha power in the Far from Threshold compared to the Near Threshold stimuli in the later time intervals.

### Moral intertrial coherence (ITC) and phase-locking value (PLV) differed in delta/theta

A significant difference was found in the Moral condition between Near Threshold and Far from Threshold in intertrial coherence (ITC).

**Figure 5:**
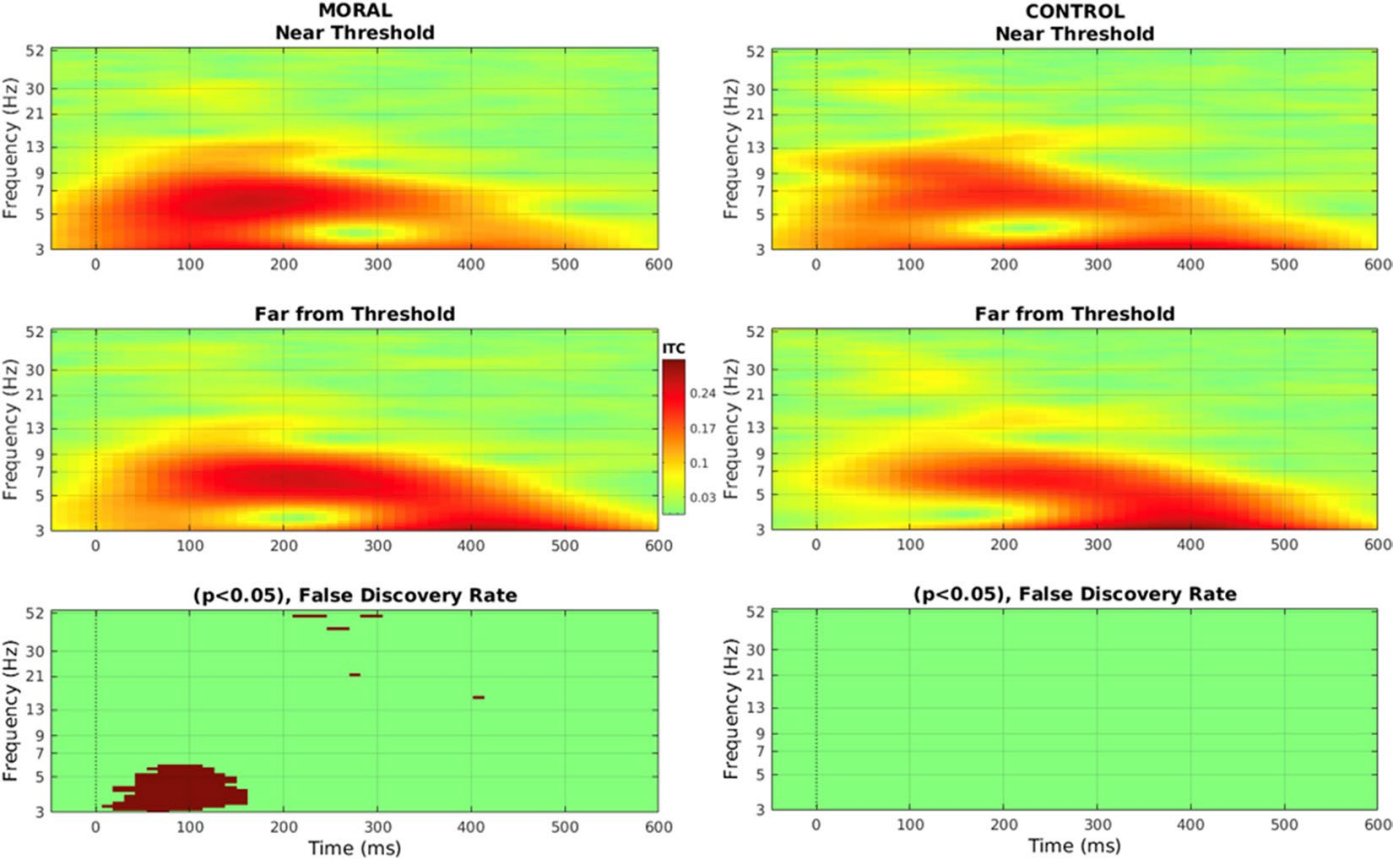
Intertrial coherence (ITC) at Pz for the first segment of the epoch (0–600 ms) in both conditions and for both proximities to threshold. ITC from baseline through stimulus onset (0 ms) to 600 ms for Moral (left side) and Control (right side) Near Threshold (top row) and Far from Threshold (second row). There was a significant difference in ITC from 3–6 Hz in the first 150 ms in the Moral condition. This was not seen in the Control condition. The significance level is 0.05, and *t*-tests are corrected for multiple comparisons using the False Discovery Rate.

**Figure 6:**
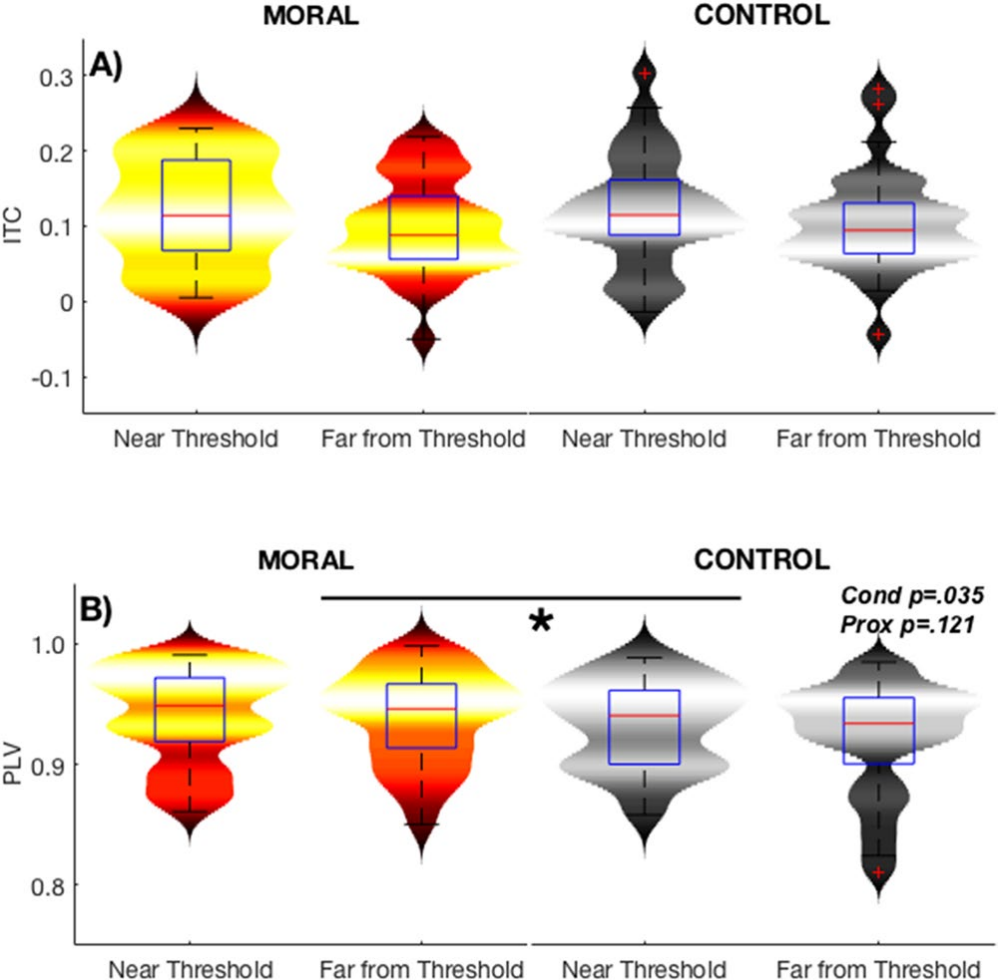
Intertrial coherence (ITC) and theta phase-locking value (PLV). (**A**) A subset of ITC data, from 3–5 Hz and between 0–100 ms for both conditions and both proximities to threshold. This is for illustration of the data distribution only, therefore there are no statistics here as they were done previously in Fig. 5. (**B**) Theta (4–7 Hz) PLV for the whole epoch (0–2000 ms) in both conditions and both proximities to threshold. In the Theta band, there was a significant effect of condition (*p*-value stated) but not of proximity to threshold.

A paired-sample *t*-test, with the False Discovery Rate correction and a significance level of 0.05, showed a significant difference between 3–6.1 Hz from stimulus onset to 160 ms (Figs 5 and 6A), with increased ITC in stimuli Near the Threshold compared to Far from the Threshold. These differences were in the delta (1–4 Hz) and theta (4–7 Hz) band. Though these differences were in the slower frequency bands and the number of cycles was low, no such differences were seen in the Control condition.

After finding significant differences in the delta and theta bands of the ITC, the phase-locking value (PLV) for only these two bands were calculated to support the ITC findings. A significant difference in PLV was found in the theta (4–7 Hz) band for the full interval (0–2000 ms) in the Moral condition. A 2 (Moral, Control) x 2 (Near, Far) ANOVA showed a significant main effect of condition (Moral, Control) on theta PLV (*F*(1,33) = 4.948, p < 0.035) with higher phase-locking in the Moral condition, but not of proximity to threshold (Near, Far), (*F*(1,33) = 2.563, *p* < 0.121) (Fig. 6B).

Taken together, delta/theta ITC showed significant increases in the Moral Individualized stimuli while theta PLV in the whole epoch was significantly higher in the Moral condition compared to the Control condition.

### Alpha power is related to reaction times and subjectively perceived emotional distress

The LPP late time interval had a significant correlation in alpha power with reaction times and the subjectively perceived emotional distress scores only in the Moral condition.

**Figure 7:**
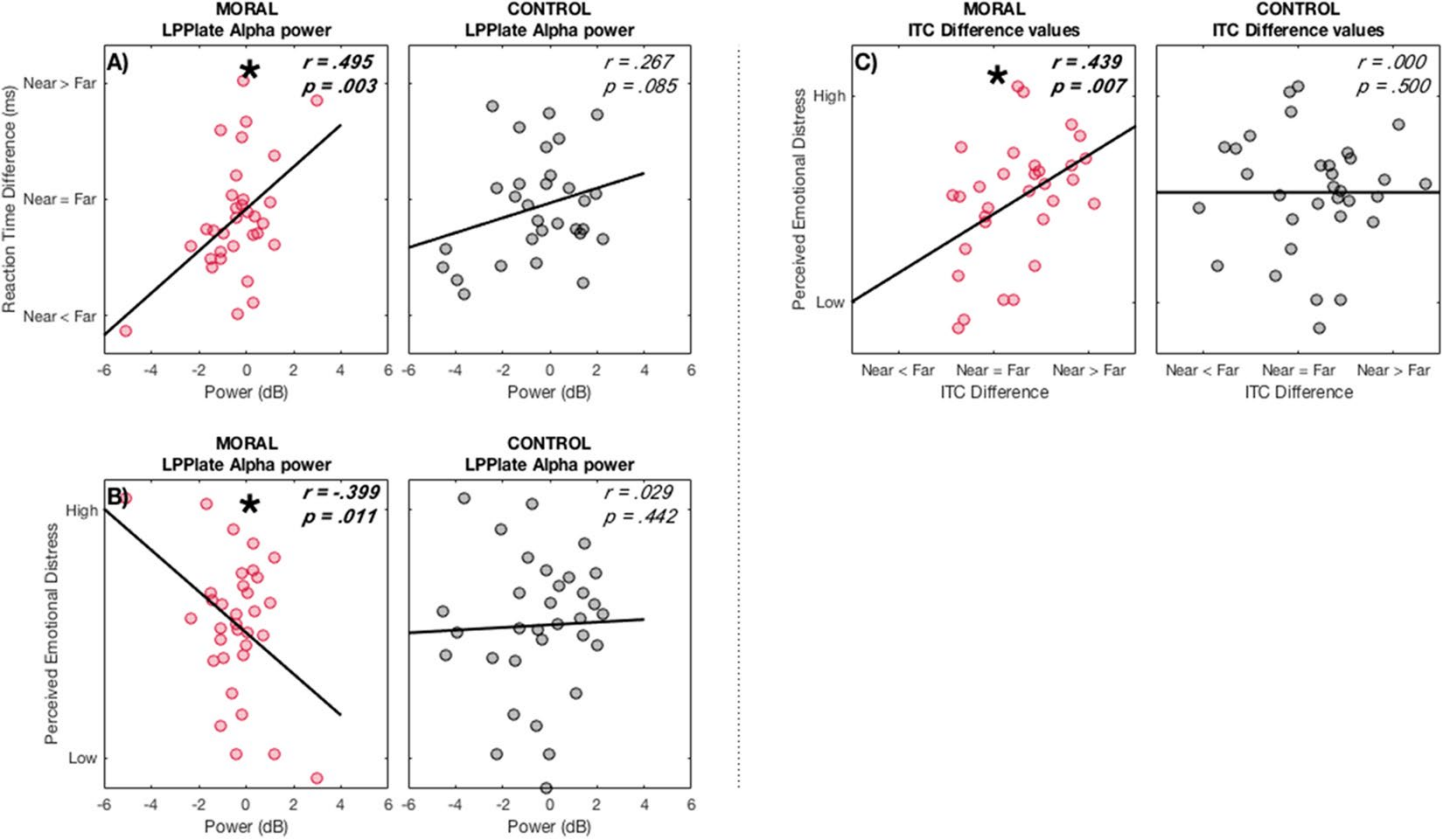
Alpha power in the LPP late time interval and early intertrial coherence (ITC) and their correlations with reaction time (RT) and perceived emotional distress scores. RT, alpha power and ITC are difference values: Near Threshold values minus Far from Threshold values. (**A**) Correlation of alpha power and RT difference in Moral and Control. The one-tailed, bootstrapped correlation was significant in the moral condition but not in the control condition. (**B**) Correlation of alpha power and perceived emotional distress scores in Moral and Control. There was a significant correlation in the Moral condition, but not in the Control condition (*r*- and *p*-values are stated). (**C**) One-tailed, bootstrapped correlations between ITC difference values and the perceived emotional distress scores. There was a significant positive correlation in the Moral condition, but not in the Control condition (*r*- and *p*-values stated). *Red*: Moral; *Black*: Control.

One-tailed bootstrapped correlations between alpha power and reaction time (*r* = 0.495, *p* < 0.003) (Fig. 7A) and the subjectively perceived emotional distress scores (*r* = −0.399, *p* < 0.011) (Fig. 7B) in the Moral condition were significant.

Taken together, in the LPP late time interval there was a significant positive relationship between Moral alpha power and reaction times and a significant negative relationship with subjectively perceived emotional distress.

### Delta/theta ITC is related to subjectively perceived emotional distress

A significant relationship between intertrial coherence (ITC) and subjectively perceived emotional distress scores was found only in the Moral condition.

The ITC values were from stimulus onset to 100 ms, between 3–5 Hz. A one-tailed bootstrapped correlation (*r* = 0.439, *p* < 0.007) was significant with a positive correlation only in the Moral condition (Fig. 7C).

In sum, Moral ITC in the delta/theta bands has a significant positive correlation with participants perceived emotional distress related to the scenarios.

## Discussion

In the present study, we investigated interindividual differences in Individualized Moral decision-making at both the behavioral and neural level. Following our three specific aims, we here report two main findings. First, our results demonstrate a central role for alpha ERSP and delta/theta phase locking – ITC and PLV - in mediating interindividual differences in Moral decision-making. Second, LPP alpha power differences between Individualized and Shared stimuli, and delta/theta ITC, all in the Moral condition, are closely related to reaction time and subjectively perceived emotional distress.

Together, our data demonstrates two main points. Firstly, interindividual differences in Moral decision-making are mediated neuronally by various markers – LPP alpha ERSP, and delta/theta ITC. Secondly, these differences are also mediated by psychological markers such as reaction time and subjectively perceived emotional distress. Our data show, for the first time, how and according to which neuronal and behavioral measures interindividual differences in Moral dilemmas can be measured.

### Interindividual differences - Late Positive Potential (LPP) and alpha power

Our results show significant differences in alpha power between Individualized stimuli near the threshold compared to Shared stimuli far from the threshold, but only in the Moral condition. As we show (Fig. 4), though alpha activity is lower in shared stimuli at an earlier time point (550 ms), this decrease in alpha lasts longer in individualized stimuli as is seen between 1000 and 1600 ms.

Consistent with the literature^40–42^, the results show that the LPP was sensitive to affective stimuli^27–32,43^ as seen in interindividual differences in perceived emotional distress (Fig. 7C). These interindividual differences were evident in alpha power, as it was linked to both behavioral measures – reaction time (Fig. 7A) – and subjectively perceived emotional distress (Fig. 7B). Despite there being, to our knowledge, only one study which investigates alpha in a Moral context^44^, it found that alpha power was significantly lower in the Moral condition compared to the non-Moral condition.

Alpha power is influenced by attention and arousal^45–48^. The interindividual nature of late alpha power seen in our data (Fig. 7A,B) may be through an elicited difference of attention. Self and attention have been shown to be related^49^ so perhaps the drive to recruit attention is highly individual in the Moral context^50^, and the decrease in alpha seen here is related to gating^51^.

Alpha has been shown to act as a gating mechanism in sensory coding for visual stimuli^52^, and for gating inhibition generally^51,53^. These previous studies, along with our findings, show that alpha power acts to gate activity in the individual related to Moral stimuli. Our results connect individualized LPP activity and inhibition through alpha power to behavioral and subjective emotional assessments. This link shows the behavioral relevance of these neuronal measures, which can illustrate differences between individuals.

### Delta/theta phase-based mechanism and subjectively perceived emotional distress

Significant differences related to stimulus proximity in delta/theta intertrial coherence (ITC) was found only in the Moral condition (Fig. 5). The difference in ITC showed that participants who had higher coherence in the near proximity in the Moral condition were more distressed when reading the scenarios than those that showed no difference (Fig. 7C). Since ITC measures phase consistency across trials, participants that were more consistent in phase coherence had more perceived distressed at the scenarios. The early emergence of this difference in ITC, before 200 ms (Fig. 6A), is consistent with the findings of implicit emotional assessments^54^.

To build on this phase consistency measure, PLV measures synchrony between channels using phase, which here centered on Pz. In PLV, a significant difference between Moral and Control was seen for the whole epoch in the theta band (Fig. 6B). The increased coherence in delta/theta relates to decision-making and memory^55–57^, attention^38^, and increases in theta spectral power associated with emotional visual stimuli^54,58–60^.

ITC phase-related interindividual differences emerge quickly and are brief, in contrast to the longer duration of that seen in the alpha results. This was shown by the significant correlation between early ITC and subjectively perceived emotional distress (Fig. 7C), which suggests that phase alignment to the stimulus is highly individual. This correlation linked this neuronal measure to a behavioral one, thus supporting its behavioral relevance.

To build on the inhibition of alpha power, event-related theta phase activity has been connected to emotional visual stimuli and emotional performance^39^. It has previously been shown that phase-related neural synchrony is involved in information processing, as is alpha power^61^. For the first time, it is shown that phase is modulated by Moral dilemmas. The results stated here isolate those changes to the delta/theta frequency range and the early part of the epoch, in the case of ITC.

Therefore, phase alignment in the early part of the epoch relates to Moral reasoning and interindividual perceived emotional distress related to the Moral dilemmas.

### Limitations

There are several limitations to this study. To begin with, there was no direct assessment of emotion or emotional valence related to the Moral scenarios. The subjectively perceived emotional distress scores were self-reported questions done after the EEG session which asked how distressing the participant found each scenario. Many studies have examined Moral dilemmas^8,9,22,34,62,63^ and the LPP^27–32,43^ related to emotion previously. Our intention was not to replicate emotion-related results, but rather look at the difference between participants on how they perceived their emotional distress related to interindividual differences at the neuronal level.

A second limitation is a lack of Control for threshold proximity. The two individualized stimuli were also the ones near the threshold. Due to the design of the study, and the aim to individualize half the stimuli to each participant, it was not possible to have two stimuli that were near the threshold but shared by all participants. The threshold itself was individualized.

The next limitation relates to the duration of the stimulus. The stimuli were presented for 2 seconds. This was short enough to prevent mind-wandering and the counting of people in the stimulus, but long enough to perceive the ratio of people killed to saved. When analysing the data, it was not possible to get reliable data for the lower frequencies (low delta) since there were less than two cycles for frequencies below 1 Hz. For the PLV analysis specifically, having longer stimulus presentation would have allowed for more cycles of lower frequencies to be measured. Therefore, differences in these low frequency ranges would have been more apparent with longer stimulus duration.

Finally, the low number of scenarios presented compared to the high repetition of stimuli is the final limitation. Since the study was event-related, and we aimed to measure difference in ERP’s, ERSP’s and ITC related to these stimuli, many trials were needed. Though this may have led to habituation to the stimuli, there were still measurable differences related to condition.

In addition to this, to maximize consistency between scenarios – consistency of scenario structure and its components – only three scenarios were presented across the whole study. This was done to minimize the risk of introducing any confounding factors by additional scenarios. Specifically, if we used many scenarios with small differences between them, the participants’ neural activity when presented with them could be different; significant aspects of the data could be lost when all trials were averaged over all scenarios. To minimize this, only scenarios which were Footbridge-type dilemmas with somewhat ecological validity, which could have the numbers of people involved made ambiguous and had a similar structure of dilemma and resulting action, were chosen from previously published studies. After employing the resulting scenarios in a pilot study, only the included scenarios were deemed ecologically valid by the pilot participants.

### Conclusions

We investigated interindividual differences in individualized Moral decision-making at both the behavioral and neural level. It was shown that there is a central role for alpha ERSP and delta/theta phase locking in mediating interindividual differences in Moral decision-making. Finally, LPP alpha power, and delta/theta ITC are closely related to reaction time and subjectively perceived emotional distress in the Moral condition.

Taken together, our data demonstrates that interindividual differences in Moral decision-making are mediated neuronally by various markers – LPP alpha ERSP, and delta/theta ITC - as well as psychologically by reaction time and subjectively perceived emotional distress. The alpha power individual differences related to Moral reasoning arise late in the epoch, mostly after 1000 ms. Phase differences related to Moral reasoning and interindividual perceived emotional distress, on the other hand, arise in the early part of the epoch.

## Methods

### Participants

Forty-one participants (mean age 30.59 years, range from 18 to 55 years; twenty-one female) completed all aspects of this study. The experimental protocols were approved by the research ethics committee of the University of Ottawa Institute of Mental Health Research (REB # 2009018), and the study was carried out with their permission. Written informed consent was obtained from each participant prior to study participation. In addition, all aspects of the experiment were performed according to the relevant guidelines and regulations of the University of Ottawa and its associated research institute.

All participants completed the Edinburgh Handedness Tool to determine handedness^64^, the Depression Anxiety and Stress Scale 42 (DASS-42)^65^ to rule out symptoms of depression and anxiety, and the Triarchic Psychopathy Measure (TriPM)^66^ to rule out psychopathic personality traits which have been shown to influence Moral decision-making^67–70^. Inclusion criteria were the following: age between eighteen and fifty-five; right-handed as per the Edinburgh Handedness Tool^64^; a Body-Mass Index between 18.0 and 30.0^71–73^; perfect or corrected-to-perfect vision.

The data from three participants was omitted from analysis due to TriPM and DASS-42 Scores that were outside the acceptable range (see Sup Mats). Finally, all participants were required to give a urine sample for a drug test using the Integrated E-Z Split Key Cup 5. This test screened participants for cocaine, methamphetamine, amphetamine, marijuana, and opiates. Two participants tested positive for marijuana; their data was removed from the study and not analyzed. In addition, the data from two participants were of poor quality due to technical issues during the recording session. Their data was excluded from all subsequent analysis. Therefore, the analyzed data that follows is from thirty-four participants.

### Behavioral Session Part 1: Varying the degree of consequentialism

Prior to the EEG session, one behavioral session was completed by each participant to determine their behavioral consequentialist threshold (Fig. 1A, Sup Fig. 1). This behavioral threshold is the maximum ratio of people killed to people saved in the scenario to which each participant assented at least 80% of the time. Determining the individual threshold of each participant allowed the individualization of these stimuli for the EEG session.

Participants were presented with a Footbridge-type scenario in paragraph form (Fig. 1B, Sup Table 1). Only Footbridge-type scenarios were chosen for the following reasons: they have been shown to increase relative brain activity in areas associated with emotional processing^17,18^ and have elicited higher self-reported emotional intensities^8^ when compared to the Trolley dilemma. Since previous studies have shown that emotion is involved in decision-making of consequential Moral dilemmas^8,9,63^, and our aim was to examine the interindividual thresholds related to these Moral dilemmas, the Footbridge scenario was used exclusively as varying the numbers of killed to saved was possible as a way of varying the consequences of the action taken.

The scenarios presented were adapted from previous studies^15,17^. Specifically, the adjustment was to generalize the number of bystanders and victims in each Moral dilemma. The purpose of the behavioral session was to determine the maximum acceptable ratio of people killed to people saved in the scenario for each participant. Since we were varying the number of bystanders sacrificed and the number of victims saved, the text was changed from specific numbers to ‘several’, ‘some’, and so on. This was the focus in this study so it was important that the numbers involved remain ambiguous. Also, our study required event-related analysis; many trials were required for ERP, ERSP and ITC analysis. To decrease the variability of neural activity between scenarios, only a small number of scenarios (two in the EEG session) were presented to maintain a consistent scenario structure, allow for ambiguous numbers of people, and maintain some believability of the scenario.

The stimuli presented during the behavioral and EEG sessions were composed of twelve two-dimensional stick-people on the left and right side of the screen, with a white line and a fixation cross down the middle separating both sides (Fig. 1). As stated in the instructions, the number of people on the left side of the screen represent the number of people that are killed in the scenario presented, and the number on the right side denote the number of people that are saved in the scenario, because of the others dying.

The task of the participant was to decide whether the ratio presented was acceptable to them. For example, when presented with two people on the left side of the screen and ten on the right side, the participant must decide if killing two people - in the scenario that they had just finished reading - to save ten people is acceptable to them. Each stimulus was presented for 2 seconds. Their response took the form of either a YES or NO, with the left and right arrow key being counterbalanced across participants as to which constituted a YES response.

The behavioral session was comprised of 110 randomly ordered trials: 10 repetitions of each stimulus. All stimuli included twelve two-dimensional people, divided between the right and left side of the screen. Also, to maintain consistency, the inverse ratios were flipped images of each other. For example, the stimulus of 5:7 was the flipped image of the stimulus of 7:5, and so on. For the 6:6 stimulus, half of the trials had the original image, the other half had its flipped image.

Between the presentation of each stimulus, a fixation cross was presented (Fig. 1B). This fixation cross, or intertrial interval (ITI), had a jittered duration of 5000 ms, 5500 ms or 6000 ms, with equal numbers of the three durations in each block. The minimum duration of the ITI was calculated based on reaction times of the pilot study (see Sup Mats for details).

### Behavioral Session Part 2: Detection of individual threshold

From the responses of the behavioral session, the consequentialist threshold of each participant was determined. To calculate this, two psychometric sigmoid functions were fit to the behavioral session data, one to the YES responses and one to the NO responses. These best-fit functions were calculated using the *glmfit* function in MATLAB. The point at which these two functions cross was determined to be their threshold (Fig. 1A-3, Sup Fig. 1) (see Sup Mats for details on the validation method).

The distribution of thresholds showed variability across participants (Fig. 2A), with the distribution negatively skewed towards the more consequential thresholds. The largest group had the most consequential threshold (the difference between people saved and killed was small, while less consequential in this instance would be that the differences would be large), 5:7, though the numbers were roughly even in the other, less consequentialist thresholds.

This is consistent with previous studies, though these studies have looked at the effect of intoxication^74^, psychopathy^69,70^, or personal versus impersonal scenarios^7^ on level of consequential responses.

### EEG Acquisition

EEG recordings were made using a 64-channel Quik-Cap (Compumedics, Charlotte, NC, USA) and were completed between 10:00 am and 6:00 pm. The channels on the Quik-Cap included: Fp1, Fpz, Fp2, Af3, Af4, F7, F5, F3, F1, Fz, F2, F4, F6, F8, FT7, FC5, FC3, FC1, FCz, FC2, FC4, FC6, FT8, T7, C5, C3, C1, Cz, C2, C4, C6, T8, TP7, CP5, CP3, CP1, CPz, CP2, CP4, CP6, TP8, P7, P5, P3, P1, Pz, P2, P4, P6, P8, PO7, PO3, POz, PO4, PO8, O1, Oz, O2. Additional channels were added for offline referencing, Independent Component Analysis (ICA) decomposition, and additional data: right and left mastoids, vertical ocular (above and below the left eye), and horizontal ocular (the outer canthi of the right and left eyes). The impedance of all channels was measured at less than 5 kΩ before recording was initiated. Data was recorded at 1000 Hz. During analysis, all files were re-referenced to the average of the two (left, right) mastoids in accordance with previous studies with a similar number of electrodes^8,23,34,35,75^.

### EEG Session

With the determination of their threshold, in the EEG session participants were presented with only four stimuli: two shared by all participants - Far from Threshold, 1:11 and 10:2 - and two individualized - Near Threshold (Fig. 1A-4). Also, as per the method mentioned above, all participants were presented with two stimuli to which they had responded YES more than 80% of the time – 1:11 and Below Threshold – and two stimuli to which they had responded NO more than 80% of the time – Above Threshold and 10:2.

The EEG session was identical to the behavioral session, with two important differences: (1) the participant was only presented with four stimuli as per their behavioral session results mentioned above; and (2) each scenario had two blocks of 60 trials, with each stimulus repeated 15 times per block (Fig. 1B). This session consisted of two scenarios (Sup Table 1); the order of scenarios was counter-balanced across participants.

For Control blocks, the same stimuli were presented, though the task was different. The participant was to judge whether there were more people on the left-hand side of the screen than the right-hand side. The same number of trials, same ITI, and same scenarios were presented before the beginning of the trials; all that differed were the instructions. In addition, there was no significant difference in duration between the Moral and the Control blocks. The order of these non-Moral cognitive Control blocks or Moral blocks was also counter-balanced across participants; participants with an odd number did the Moral then Control blocks, while those with an even number did the Control then Moral blocks.

Participants were seated in a dark, quiet room, between 55–60 cm away from the computer screen, as per their comfort. The experimental paradigm was presented to the participant using E-Prime 2.0 software (Psychology Software Tools, Inc., Sharpsburg, PA, USA). The EEG data was recorded with no high-pass, low-pass or notch filters at a sampling rate of 1000 Hz and referenced online to the right mastoid (*data is available from A Wolff, the corresponding author*).

### Event-related potential (ERP) Data analysis

All EEG data preprocessing and analysis of the event-related potentials (ERPs) were completed using EEGLAB (versions 12,13)^76^, which required MATLAB version 2014a or 2016a, including the use of the Optimization and Signal Processing Toolboxes. All statistical analysis (except for the ERSP and ITC) was completed using SPSS 24.

For preprocessing, data - recorded at 1000 Hz - was resampled to 500 Hz in EEGLAB using MATLAB’s *resample* function. The reason for this was purely practical, to reduce the size of the files for storage purposes. Since the highest frequency being investigated was 55 Hz (in ERSP and ITC analysis), the decreased sampling rate was well above the Nyquist frequency^77^. The continuous data was then high-pass and low-pass filtered from 0.5 Hz to 30 Hz for ERP analysis according to previous studies^12,13,23,26,44^, and from 0.5 to 55 Hz for ERSP, ITC and PLV analysis in EEGLAB using FIR filtering (just below the 60 Hz line noise).

The participants’ data was then visually inspected, and epoched with a baseline of −200 ms (approximately two alpha cycles) to stimulus onset. All artifacts, specifically blinks and saccades, were reduced using independent component analysis (ICA) and the Multiple Artifact Rejection Algorithm (MARA)^78,79^ of EEGLAB which standardized the artifact rejection process.

Participants all had a minimum of 12 clean trials per block, therefore 48–60 clean trials per stimulus per condition were included in the analysis. When Individualized stimuli was compared to Shared stimuli, two stimuli were grouped together (see Fig. 1A-4). Therefore, the number of trials in this grouping was 96–120.

For each ERP component, the activity of one electrode was examined. The breakdown for each component was as follows: for N100, P300 and the LPP, Pz was the electrode chosen, and for the N200, Cz was chosen according to previous studies^12,16,32,33,35,42,75,80–82^. The electrode sites and time windows for these components were selected according to the literature^16,32,75,80–84^ and visual inspection of the ERP grand average waveforms for both conditions and groups of stimuli.

The time intervals for each component, as measured in previous studies, was as follows: for the N100, 100–220 ms^80^; N200, 250–350 ms^75^; P300, 350–450 ms^16^. For the Late-Positive Potential (LPP), the early phase (400–1000 ms) and the late phase (1000–2000 ms) was measured^32,42,81,82^ (Fig. 3B). The maximum amplitude was measured for the N100, N200, and P300^16,75,80^, while the mean amplitude was measured in the LPP due to the long time intervals and the fact that this was the measurement used in previous studies^32,82,83^.

To determine if there was a difference in activity related to the response, an ERP at Pz was time-locked to the response in each trial for all stimuli in Moral and Control (Sup Fig. 3). A repeated measures *t*-test found no significant difference between the conditions, using the False Discovery Rate to account for multiple comparisons (Sup Fig. 3).

### Event-Related Spectral Perturbation (ERSP) analysis

For Event-Related Spectral Perturbation (ERSP) analysis, all preprocessing and epoching steps were identical to that of the ERP data, except that the data was low-pass filtered at 55 Hz in EEGLAB using FIR filtering. Statistical differences between stimuli were measured for both conditions using EEGLAB’s statistics and using the False Discovery Rate to account for multiple comparisons, at a significance level of 0.05 (Fig. 4). A 3-cycle (window length of 0.8 s) Morlet wavelet analysis (linear scale) was employed, with a Hanning tapered window and the Gaussian wavelet at 7 Hz (results shown begin at 3 Hz).

Differences in ERSP were investigated during one large time interval which comprised the intervals of the P300 and LPP (the results were illustrated from 400–2000 ms in Fig. 4 since there were no significant differences in the early part of the P300 time interval). As was the case in the ERP analysis, the ERSP was only measured at Pz since this is where the ERP findings were, and after visual inspection of the topographical maps for each condition and groups of stimuli.

To determine if there was a difference in activity related to the response, an ERSP at Pz was time-locked to the response for all stimuli in Moral and Control (Sup Fig. 4). The response for each trial was the event to which the ERSP was time-locked. A repeated measures t-test found no significant difference between the conditions, using the False Discovery Rate to account for multiple comparisons (Sup Fig. 4).

### Intertrial coherence (ITC) analysis

Intertrial Coherence (ITC) was calculated in EEGLAB^76^ using the same data as for the ERSP analysis. The time interval of the entire epoch was measured, from stimulus onset to 2000 ms (though Fig. 5 only shows the first 600 ms of the epoch since that was where the significant differences were measured).

Statistical differences between stimuli were measured for both conditions using EEGLAB’s statistics, at a significance level of 0.05, with the False Discovery Rate to account for multiple comparisons (Fig. 5).

### Phase-locking value (PLV) analysis

PLV was calculated in Brainstorm^85^ using the same data from the ERSP and ITC analysis. Two frequency bands were measured, Delta (1–4 Hz) and Theta (4–7 Hz), and the time interval constituted the entire epoch, from stimulus onset to 2000 ms. Since the ERSP and ITC were investigated only at electrode Pz, the PLV used Pz as the central electrode, with the phase-locking between Pz and four of its surrounding electrodes – P1, CPz, POz, and P2 – calculated. The mean of these four values, namely phase-locking between Pz and P1, Pz and CPz, Pz and P2, and Pz and POz, was calculated, and it was this mean value that was included in the statistical analysis.

### Post-session questionnaire emotion scores

Once the EEG session had been completed, each participant responded to a question related to the three scenarios presented in the behavioral and EEG session. For each of the three scenarios, one question was asked relating to the participants’ emotional assessment of each scenario. Participants responded on a visual-analog scale, and the mean of the three values (for the three scenarios) was calculated to have one value with which to correlate the neural data, thus reducing the number of correlations (see Sup Mats for more information).

## Supplementary information

Supplementary Materials
