# Supplementary material for: Interindividual neural differences in moral decision-making are mediated by alpha power and delta/theta phase coherence
Source: Sci Rep. 2019 Mar 14;9:4432. doi: 10.1038/s41598-019-40743-y (PMC6418194; doi:10.1038/s41598-019-40743-y)

**­­­Title:** Interindividual neural differences in moral decision-making are mediated by alpha power and delta/theta phase coherence

**Authors:**

*Annemarie Wolff ^1^ University of Ottawa Institute of Mental Health Research

1145 Carling avenue

Ottawa, ON Canada

K1Z 7K4

[awolf037@uottawa.ca](mailto:awolf037@uottawa.ca)

(613) 798-6963

Javier Gomez-Pilar ^2^

Takashi Nakao ^3^

Georg Northoff ^1^

**Affiliations:**

*^1^ Institute of Mental Health Research, University of Ottawa, Ottawa, Canada*

*^2^ Biomedical Engineering Group, Higher Technical School of Telecommunications Engineering, University of Valladolid, Valladolid, Spain*

*^3^ Department of Psychology, Graduate School of Education, Hiroshima University, Hiroshima, Japan*

***SUPPLEMENTARY MATERIALS***

1. Methods:
   1. *Participants*

The DASS-42 shows a high correlation with the Beck Anxiety and the Beck Depression Inventories (.81 and .74 respectively) ^1^. The TriPM was administered as it was designed for the general population and is self-report. It has three sub-scales - Boldness, Meanness and Disinhibition - that are considered to be a comprehensive view of the psychopathic personality ^2–4^.

Exclusion criteria was the following: history of psychiatric or neurological illness; history of head injury; current or history of substance abuse, including alcohol; history of radiation therapy to the head or neck; history of electroconvulsive therapy; TriPM Meanness and Inhibition scores greater than 15; DASS-42 scores for depression greater than 14, anxiety greater than 10, or stress greater than 19.

The purpose of the drug test was to rule out the presence of substances which would alter the EEG data ^5,6^.

- 1. *Behavioral Data Analysis*

Responses and reaction times were analyzed from the E-Prime 2.0 log files for each block of the experiment. This data was analyzed in either MATLAB 2014a or 2016b, and this data was transferred to SPSS 24 for statistical analysis.

- 1. *Behavioral Session Part 1: Varying the degree of consequentialism*

The scenario in the behavioral session and those in the EEG session (Sup Table 1) had roughly the same number of characters (451-452), and identical paragraph and sentence structures. Once the participants had completed reading the scenario at their own pace, they were given instructions for the task.

The minimum duration of the intertrial interval (ITI) was calculated from a behavioral pilot study which was completed in advance of this EEG study. Ten participants completed the behavioral session as structured here, and the mean reaction time of each participant was calculated. To determine the minimum ITI, the maximum reaction time from this pilot study was tripled. This maximum reaction time was 1.63 seconds, so tripled its duration was 4.89 seconds. The closest whole number was 5.0, so 5.0 seconds was the minimum ITI, with the other two ITI’s increasing by half a second each.

- 1. *Behavioral Session Part 2: Detection of individual threshold*

The validation method involved determining between which two stimuli did the participant’s YES response rate drop from greater than 80% to less than 80%. These two stimuli would then constitute those below and above the threshold. For example, one participant responded YES to 1:11 at a rate of 100%, 100% to 2:10, 90% to 3:9, 20% to 4:8, 5% to 5:7, and 0% to 6:6. According to this second method, the participant’s threshold is between 3:9 and 4:8. The primary (sigmoid functions fit to the response data in the behavioral session) and verification methods were computed independently, and the same threshold was determined using both methods for all participants.

- 1. *Post-Session Questionnaire Emotion scores*

A 10cm horizontal line, on which the participant was directed to make a vertical mark to indicate where on the line their response fell, was presented for each question on which the participant was to respond. The vertical marks of each participant were scored in the following way: a value of zero was at the 5cm, or halfway point, between the two extremes. The more to the left of the zero, the more the negative score was given, with a maximum of -5.0. The same was true to the right of the zero; the maximum was +5.0, and the more to the right of the zero, the higher the score.

- 1. *Event-related potential (ERP) Data Analysis*

EEG data preprocessing was done using EEGLAB versions 12-13 ^7^ which worked with MATLAB version 2016a. For each stimulus for each participant, all trials were averaged. Near Threshold was comprised of Below Threshold and Above Threshold stimuli, and Far from Threshold were comprised of 1:11 and 10:2 stimuli.

1. Results:
   1. *Behavioral data*

From the behavioral session, it was determined that the distribution of participants’ threshold was the following: five participants had a maximum ratio of killing one person to save eleven (1:11); five participants had a maximum ratio of 2:10; four participants had a maximum ratio of 3:9; five had a maximum ratio of 4:8; fifteen had a maximum ratio of 5:7 (Fig 2A).

In the EEG recording sessions, the reaction times of all Moral trials were compared with the reaction times of all Control trials to determine if there was a significant effect of condition, regardless of stimulus. The distributions of all trials of both conditions were found to be non-normal (Moral - Kolmogorov-Smirnov p=.000, Control - Kolmogorov-Smirnov p=.000), so a nonparametric related-samples test was carried out.

Overall, the moral condition showed significantly slower reaction times, as did stimuli that were near the threshold, when compared to control and stimuli far from the threshold. Also, the difference in reaction times was retained when differences between the thresholds themselves was measured; the effect of condition on reaction times differed also, as is shown by a significant interaction.

- 1. *Post-Session Questionnaire Emotion scores*

The distribution of mean emotion scores had a mean of 0.153, median of 0.4042, and standard deviation of 2.3458 (Sup Mats Fig 4C). The range of scores was from -4.98 to 4.38, and the distribution was negatively moderately skewed, skewness = -0.515.

- 1. *Event-related potentials (ERP's)*

A repeated measures ANOVA was conducted to compare the main effect of condition and stimulus on either the maximum amplitude or mean activity for the specified electrode (Fig 3). Condition consisted of two levels (Moral, Control) and proximity to threshold consisted of two levels (Near, Far).

- - 1. *Early Components*

In the early components, there was no significant main effect of Condition (Moral, Control) in the N100 (Wilks’ Lambda = .977, *F*(1,30) = .670, p = .420) or the N200 (Wilks’ Lambda = .971, *F*(1,30) = .876, p = .357) (Fig 3A). The N200 also had no significant effect of proximity to threshold (Near, Far), (Wilks’ Lambda = .982, *F*(1,30) = .531, p = .472) (Fig 3A).

- - 1. *Late Components*

In the late components, there was no significant main effect of Condition (Moral, Control) in the P300 (Wilks’ Lambda = 1.0, *F*(1,30) = .000, p = 1.000), the LPP early (Wilks’ Lambda = .928, *F*(1,30) = 2.255, p = .144), or the LPP late (Wilks’ Lambda = .991, *F*(1,30) = .272, p = .606) (Fig 3B). The LPP early also had no significant effect of proximity to threshold (Near, Far), (Wilks’ Lambda = 1.000, *F*(1,30) = .000, p = 1.000) (Fig 3B).

- 1. *Correlation between behavioral data and neural data*

To determine if there was a significant relationship between behavioral data and neural data, one-tailed correlation with mean reaction times was done. To reduce the number of correlations overall, and to further focus on the difference related to proximity to the threshold, the value of the Far from Threshold variable was subtracted from the value of the Near Threshold variable.

Two additional steps taken to strengthen the statistical basis of these correlations was first to bootstrap each correlation (1000 samples), and second to halve the significance level from 0.05 to 0.025. Finally, only the LPP time intervals (400-2000ms) in the ERSP and the early (0-100ms) were measured for their relationship with behavioral data.

The first such analysis was between the difference values for the reaction times (Near Threshold reaction time minus Far from Threshold reaction time) and the

1. *Alpha power is related to reaction times and emotion scores*

The LPP late time interval had no significant correlation in alpha power with reaction times and the emotion scores in the control condition. One-tailed bootstrapped correlations between 1) alpha power difference values (Near Threshold minus Far from Threshold) and reaction time difference values (*r* = 0.267, *p* < 0.085); and 2) alpha power difference values and the emotion scores (*r* = 0.029, *p* < 0.442) were not significant.

1. *Delta/theta ITC is related to emotion scores*

With the subset of ITC data illustrated in Fig 6A, one-tailed, bootstrapped correlations were done with the emotion scores. To reduce the number of correlations from two per condition to one, the ITC difference values were computed – ITC in Near Threshold minus ITC in Far from Threshold (Fig 7C). As above, results were significant at 0.025.

In the Moral condition, there was a significant correlation between these scores and the difference in ITC (Fig 11B). In the Control condition, in contrast, there was no significant correlation with a Pearson correlation value of *r* = 0.000, *p* < 0.500.

1. Discussion:
   1. *Behavioral Results*

Concerning reaction times, it was determined that the Moral trials had significantly longer reaction times than Control. Previous studies have shown higher reaction times in personal dilemmas compared to impersonal dilemmas ^8^, and higher reaction times when family members are involved in the personal scenario compared to the involvement of strangers ^9^. This shows a significant effect of condition on reaction time, which can be extended to a significant effect of proximity to threshold as well. The Near Threshold stimuli had significantly longer reaction times. This indicates a more cognitively demanding task and higher conflict; this would be consistent with the results found when family members were involved in the personal dilemmas ^9^. The effect was maintained when the proximity to threshold was further broken down to all four stimuli as the stimuli that were above and below the threshold had higher reaction times longer than those far from the threshold, 1:11 and 10:2.

The final behavioral measure, the threshold distribution itself, had a significant effect on reaction time, with the more consequential thresholds (5:7, 4:8, 3:9) having longer reaction times than the less consequential thresholds (1:11, 2:10). Already, the effect of condition is apparent, as is the proximity to the threshold. Since our research questions concerned variability in consequentialism and inter-individual differences - both of which are measured by the varying thresholds - the behavioral results provide the foundation on which to continue with these questions when assessing our neural data.

References:

1. Brown, T. A., Chorpita, B. F., Korotitscw, W. & Barlow, D. H. Psychometric Properties of the Depression Anxiety Stress Scales (DASS) in Clinical Samples. *Behav. Res. Ther* **35,** 79–89 (1997).

2. Sellbom, M. & Phillips, T. R. An examination of the triarchic conceptualization of psychopathy in incarcerated and nonincarcerated samples. *J. Abnorm. Psychol.* **122,** 208–214 (2013).

3. Drislane, L. Clarifying the Content Coverage of Differing Psychopathy Inventories through Reference to the Triarchic Psychopathy Measure. *imPORTANT HENT NY VERSION* **6,** 2166–2171 (2008).

4. Venables, N. C., Hall, J. R. & Patrick, C. J. Differentiating psychopathy from antisocial personality disorder: A triarchic model perspective. *Psychol. Med.* **44,** 1005–1013 (2014).

5. Banoczi, W. How some drugs affect the electroencephalogram (EEG). *Am. J. Electroneurodiagnostic Technol.* **45,** 118–129 (2005).

6. Blume, W. T. Drug effects on EEG. *J. Clin. Neurophysiol.* **23,** 306–311 (2006).

7. Delorme, A. & Makeig, S. EEGLAB: An open source toolbox for analysis of single-trial EEG dynamics. *J. Neurosci. Methods* **134,** 9–21 (2004).

8. Wang, Y., Deng, Y., Sui, D. & Tang, Y.-Y. Neural correlates of cultural differences in moral decision making. *Neuroreport* **25,** 110–6 (2014).

9. Chen, P., Qiu, J., Li, H. & Zhang, Q. Spatiotemporal cortical activation underlying dilemma decision-making: An event-related potential study. *Biol. Psychol.* **82,** 111–115 (2009).


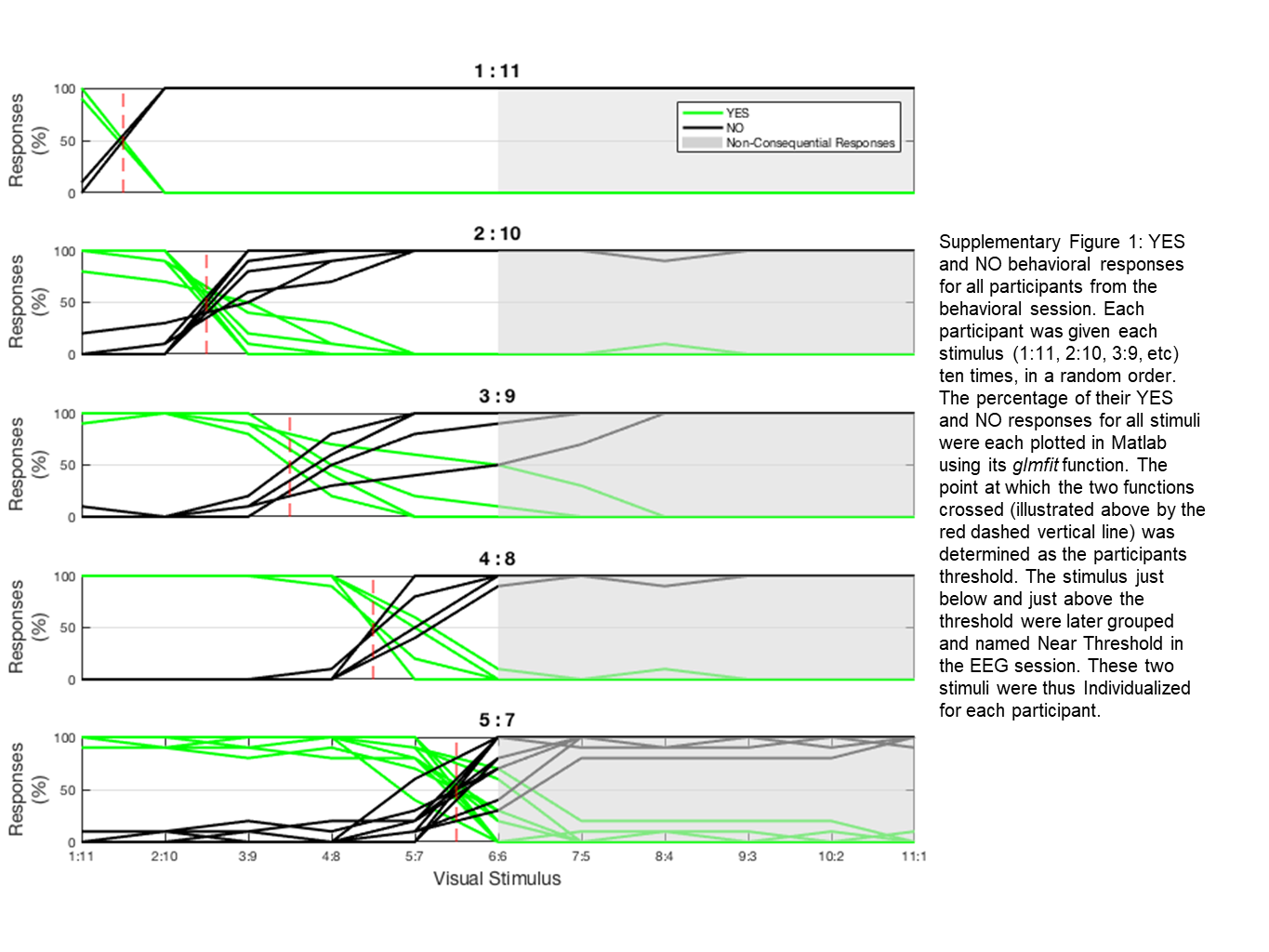


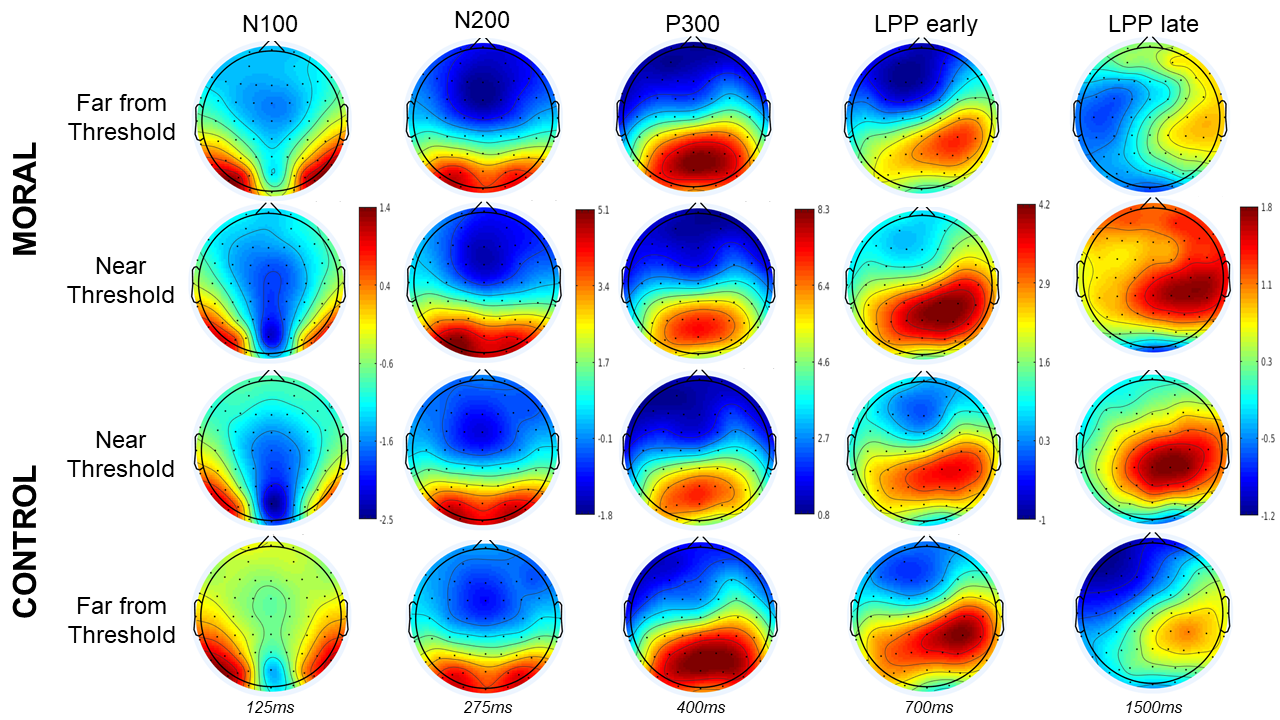


Supplementary Figure 2: Topographical maps of all four stimuli during the time interval of each component. *Rows*: stimuli, *columns*: components. The time at which each topo plot was measured is located at the bottom of each column. Colorbars are located to the right of each column.


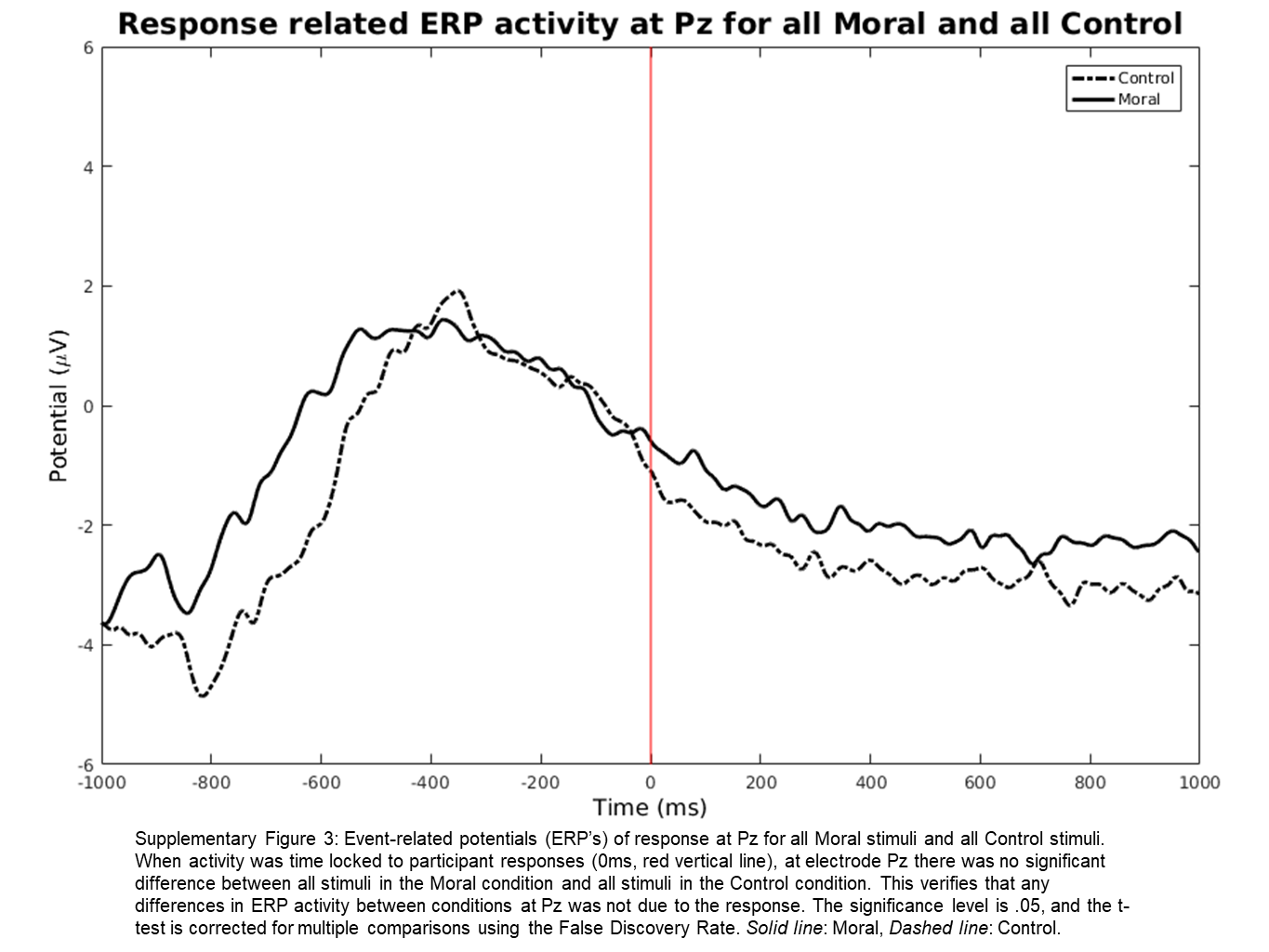


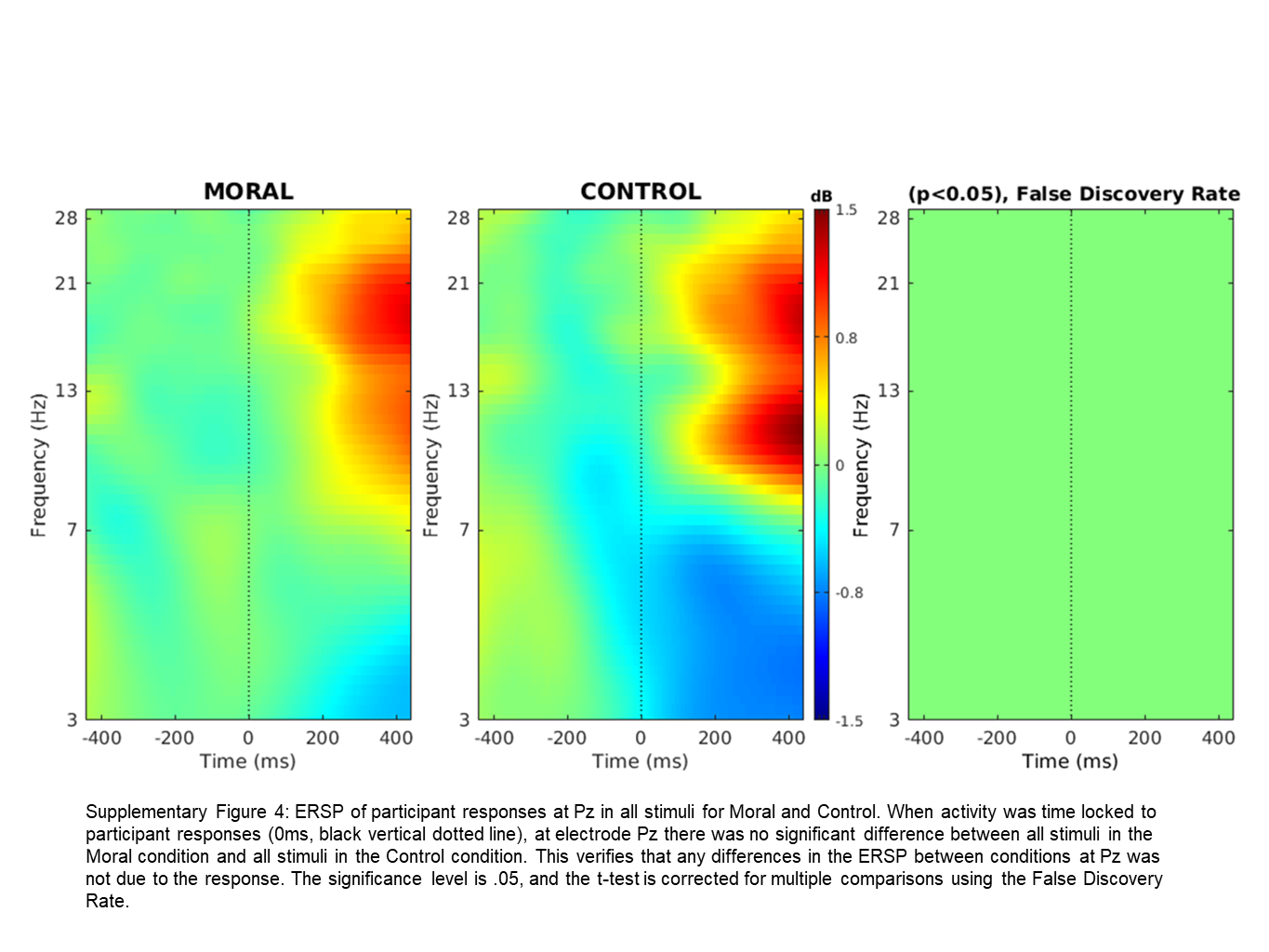


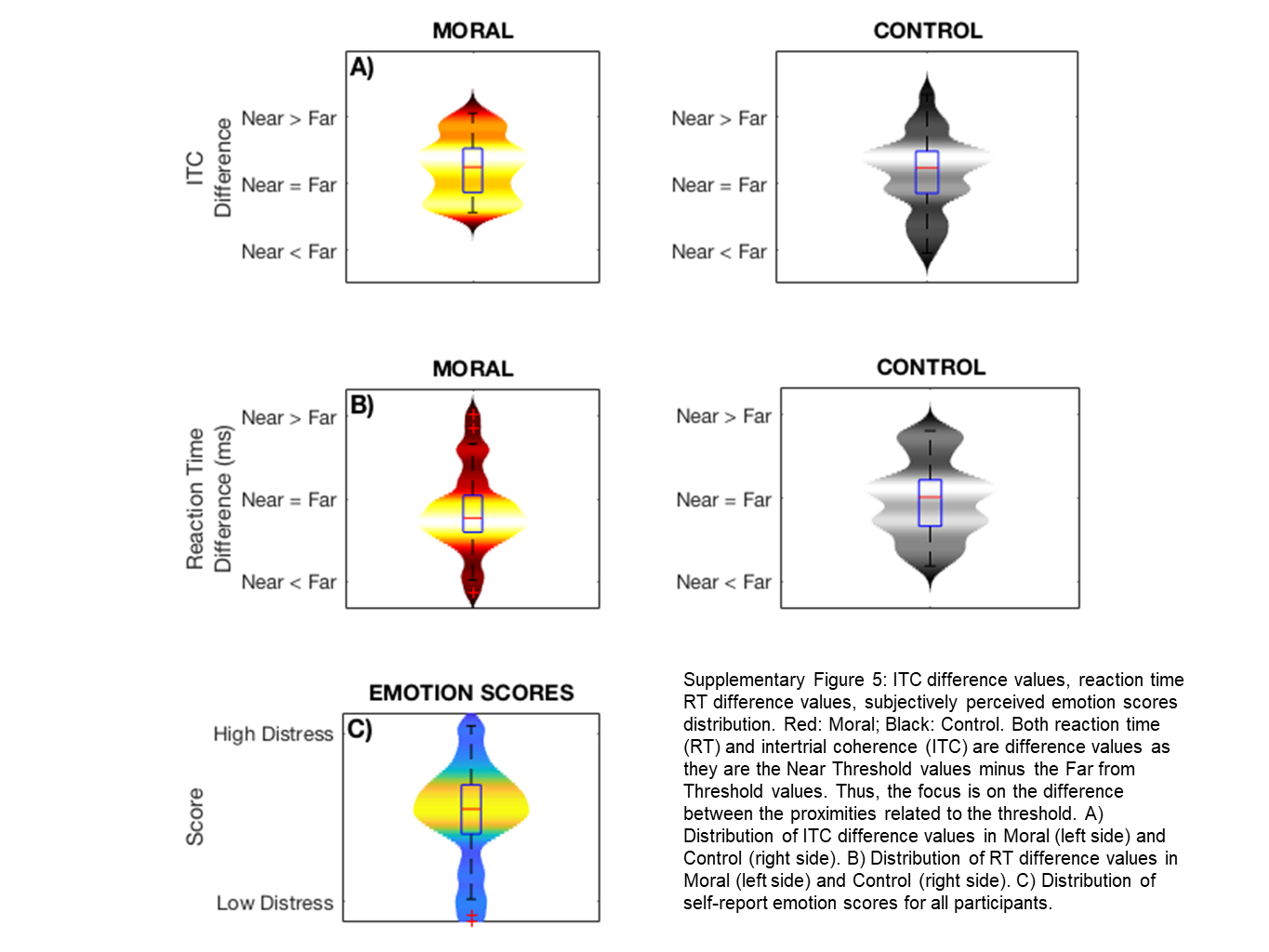

Supplement: Supplementary file 1 — Supplementary Materials [file 41598_2019_40743_MOESM1_ESM.docx]
